# Seasonal variations in use and outcome of rapid antigen detection tests and cultures in pharyngotonsillitis: a register study in primary care

**DOI:** 10.1186/s12879-021-06774-5

**Published:** 2021-10-26

**Authors:** Martin Andersson, Jon Pallon, Olof Cronberg, Martin Sundqvist, Katarina Hedin

**Affiliations:** 1 Department of Research and Development, Region Kronoberg, Växjö, Sweden; 2 Department of Clinical Sciences in Malmö, Family Medicine, Lund University, Malmö, Sweden; 3 Department of Laboratory Medicine, Clinical Microbiology, Faculty of Medicine and Health, Örebro University, Örebro, Sweden; 4 Futurum, Region Jönköping County, and Department of Health, Medicine and Caring Sciences, Linköping University, Linköping, Sweden

**Keywords:** Sore throat, Tonsillitis, Pharyngitis, Seasonal variation, *Streptococcus pyogenes*, *Streptococcus dysgalactiae*, *Fusobacterium necrophorum*

## Abstract

**Background:**

Diagnosis and treatment of pharyngotonsillitis are commonly focused on group A streptococci (GAS), although the disease is often associated with other pathogens. While the incidence of pharyngotonsillitis is known to vary with season, seasonal variations in the prevalence of potential pathogens are sparsely explored. The aim of this study was to explore any seasonal variations in the use and outcome of rapid antigen detection tests (RADTs) for GAS and throat cultures among patients diagnosed with pharyngotonsillitis in primary care.

**Methods:**

We retrieved and combined retrospective data from the electronic medical record system and the laboratory information system in Kronoberg County, Sweden. Primary care visits resulting in a diagnosis of tonsillitis or pharyngitis were included, covering the period 2013–2016. The monthly rate of visits was measured, along with the use and outcome of RADTs for GAS and throat cultures obtained on the date of diagnosis. The variations between calendar months were then analysed.

**Results:**

We found variations between calendar months, not only in the mean rate of visits resulting in a diagnosis of pharyngotonsillitis (p < 0.001), but in the mean proportion of RADTs being positive for GAS among the diagnosed (p < 0.001), and in the mean proportion of visits associated with a throat culture (p < 0.001). A lower mean rate of visits in August and September coincided with a lower proportion of RADTs being positive for GAS among them, which correlated with a higher proportion of visits associated with a throat culture.

**Conclusions:**

This study suggests that the role of GAS in pharyngotonsillitis in Sweden is less prominent in August and September than during the rest of the year.

**Supplementary Information:**

The online version contains supplementary material available at 10.1186/s12879-021-06774-5.

## Background

A sore throat is a common condition, resulting in considerable numbers of primary care visits and prescriptions of antibiotics. According to the Primary care Record of Infections in Sweden (PRIS), 11% of the prescriptions of antibiotics are issued for sore throats (the vast majority being diagnosed with either pharyngitis or tonsillitis) [1], but consultation rates and prescription percentages vary between countries [2]. The European clinical guideline for the management of acute sore throat [3], and the Swedish equivalent for pharyngotonsillitis [4], are based on the Centor scoring system [5], assessing the probability of group A streptococci (GAS) in the pharynx [6]. In the Swedish official guideline, antibiotics are only recommended to patients with three or four Centor criteria, and a positive rapid antigen detection test (RADT) for GAS [4]. Throat cultures are not recommended for routine diagnosis but can be indicated for patients with worsened or non-resolving symptoms.

GAS are the most commonly found bacteria among adolescents and adults seeking medical care for pharyngotonsillitis, but the disease has been associated with a variety of pathogens, both bacteria and viruses, including polymicrobial cases [3, 7]. Bacteria can be found among less than half of the patients seeking medical care for pharyngotonsillitis [7]. Also, asymptomatic colonization by different pathogens is prevalent [3, 7, 8].

In Sweden, with a predominantly temperate climate, pharyngotonsillitis is generally considered to be most common during the colder months [9], but seasonal variations in the prevalence of potential pathogens are sparsely explored. A better understanding of seasonal variation in aetiology could improve the quality of future diagnosis and treatment.

The aim of this study was to explore any seasonal variations in the use and outcome of rapid antigen detection tests (RADTs) for GAS and throat cultures*,* among patients diagnosed with pharyngotonsillitis in primary care.

## Methods

We retrieved and combined retrospective data from the electronic medical record (EMR) system (Cambio COSMIC, Cambio Healthcare systems, Stockholm, Sweden) and the laboratory information system (ADBAKT, Autonik, Nyköping, Sweden) in Kronoberg County, Sweden, covering the period from 2013-01-01 to 2016-12-31. All the publicly financed Primary Health Care Centres (PHCCs) in the county were asked to participate, and 31 out of 34 PHCCs agreed. Due to the system of publicly financed health care, with every citizen connected to a PHCC, our data comprised the vast majority of the population of the county. The mean population included during the 4 years studied was 177,675, while the mean population of the whole county during the period was 191,000 [10].

Visits to primary care resulting in a diagnosis of acute tonsillitis (ICD-10 J03) or acute pharyngitis (ICD-10 J02) were included and merged, as diagnostic traditions and clinical guidelines do not separate the two. If not otherwise stated, all further use of the word “visits” in this article refers to this definition. From the EMRs, we extracted the date of diagnosis, the personal identification number, if a rapid antigen detection test (RADT) for GAS had been used for that patient and date, and if used, the result of that RADT. Any results from throat cultures obtained from that patient and date, were extracted from the laboratory information system. Two types of standardized throat cultures were available at the time, one targeting beta-haemolytic streptococci (A, C and G) only, and the other one also including selective anaerobic incubation to detect *Fusobacterium necrophorum*. As to duplicates of visits (more than one visit on the same date) in our dataset, we were not able to tell if they represented real duplicates or only duplicated data transfers, as most of them were identical regarding reporting unit/PHCC, and when so, also identical regarding RADT use and results. For technical reasons, we chose to count what appeared to be the first visit, but it should be noted that it does not necessarily reflect true chronology due to data limitations. However, the number of duplicates in the dataset was low (179 of 21,363), and the true number of duplicates possibly even lower. As to duplicates of cultures, positive results were included.

In late 2013, the microbiological laboratory switched from Lancefield classification of streptococci to binomial nomenclature, with the introduction of MALDI-TOF (matrix-assisted laser desorption/ionization with time-of-flight mass spectrometer). For the analyses in this article, we chose to treat group A beta-haemolytic streptococci (GAS) and *S. pyogenes* as interchangeable, and similarly, to collectively treat group C (GCS) and G (GGS) streptococci as equivalent to *S. dysgalactiae* subsp. *equisimilis*.

## Statistics

Age quartiles were calculated, after adjusting age to the number of full years lived. Three age groups were defined, to control for age as a potentially interfering factor. These age groups were chosen due to previous knowledge of age being of relevance to aetiology [8, 11]. For descriptive statistics, the rate of visits was measured per calendar month, with the numbers weighted to the length of the month by dividing by the number of days in the month and multiplying by 30. Data from all 4 years were combined, for further analyses. Seasonal variation was first analysed with chi-squared goodness of fit tests for detection of overall differences between all calendar months (with aggregated data from all 4 years, if not stated otherwise in the text). The magnitude of any detected seasonality was then estimated with peak-to-low ratios between the months with the highest and the lowest rates or proportions, as exemplified by Christiansen et al. [12]. The confidence level was set to 95%. Confidence intervals for proportions were calculated using Wilson’s method. Confidence intervals for ratios (ratios of proportions) were calculated according to Daly, 1998, with p-values from z-tests according to Sheskin, 2004. Correlations were analysed using Pearson’s bivariate correlation. The analyses were performed using Excel, version 16.16.2 (Microsoft, Redmond, WA) and SPSS, version 23.0.0.0 (IBM, Armonk, NY).

## Results

### Visits with a diagnosis of pharyngotonsillitis

**Fig. 1:**
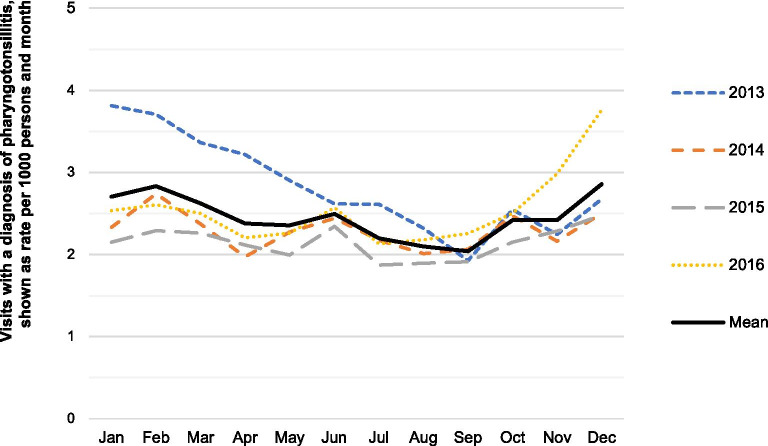
Visits to primary care with a diagnosis of pharyngotonsillitis 2013–2016

Throughout the 4 years of the study, a total of 21,184 visits with a diagnosis of tonsillitis or pharyngitis were identified, after exclusion of 179 duplicates (more than one visit on the same date). The median age was 20 years. Of all visits, 56% (95% CI 56–57) were made by men. However, this gender difference was not seen in the youngest age group (0–14 years). The rate of visits per month showed seasonal variation when comparing means for all calendar months (p < 0.001). The highest mean was seen in December, and the lowest in September, with a peak-to-low rate ratio of 1.40 (95% CI 1.22–1.60, p < 0.001). The second lowest mean was seen in August, with a corresponding rate ratio (December/August) of 1.36 (95% CI 1.19–1.55, p < 0.001). However, peak and low months were not the same for all the years studied (Fig. 1). For instance, the beginning of 2013 and the end of 2016 had higher numbers, thus skewing the mean. In the age group of 15–29 years, the mean pattern was somewhat different, with a peak in August. Data for all years are shown in Table 1, including rate ratios between peak and low months. Summarized characteristics per calendar month are presented in Tables 2, 3, 4, 5. Non-aggregated data are also shown in Additional file 1: Table 1.

**Table 1:** Visits with a diagnosis of pharyngotonsillitis 2013–2016, shown as rate per 1000 persons and month

	Jan	Feb	Mar	Apr	May	Jun	Jul	Aug	Sep	Oct	Nov	Dec	p^a^	RR peak/low (95% CI), p^b^
2013	3.81	3.71	3.36	3.22	2.90	2.62	2.61	2.32	1.93	2.55	2.24	2.67	< 0.001	1.98 (1.74–2.26), p < 0.001
2014	2.33	2.74	2.37	1.97	2.27	2.44	2.17	2.01	2.07	2.47	2.16	2.50	< 0.001	1.39 (1.21–1.59), p < 0.001
2015	2.15	2.29	2.26	2.12	1.99	2.35	1.87	1.89	1.91	2.15	2.28	2.48	< 0.001	1.32 (1.15–1.53), p < 0.001
2016	2.54	2.60	2.50	2.20	2.26	2.57	2.13	2.18	2.26	2.50	2.98	3.76	< 0.001	1.76 (1.56–2.00), p < 0.001
Mean	2.71	2.83	2.62	2.38	2.36	2.49	2.20	2.10	2.04	2.42	2.42	2.86	< 0.001	1.40 (1.22–1.60), p < 0.001

All ages. The numbers are weighted to the length of the month (divided by the number of days and multiplied by 30)

*CI* confidence interval

^a^P-value from chi-squared goodness of fit test for overall differences in rates between all months

^b^Rate ratio between the month with the highest rate and the month with the lowest rate, and corresponding p-value from z-test (Sheskin, 2004)

**Table 2:** Summary of visits with a diagnosis of pharyngotonsillitis, per calendar month (2013–2016 combined). All ages

	Jan	Feb	Mar	Apr	May	Jun	Jul	Aug	Sep	Oct	Nov	Dec	$\overline{x }$
Mean number of weighted visits*	479	502	465	422	419	443	390	374	363	430	431	510	436
Median age, years (IQR)	20 (37–8)	19 (35–7)	18 (35–7)	20 (36–8)	19 (36–8)	20 (36–9)	21 (37–9)	21 (36–11)	19 (34–10)	20 (34–8)	18 (32–7)	19 (35–8)	20 (35–8)
Women, % of visits (95% CI)	46 (44–48)	46 (43–48)	42 (40–44)	43 (40–45)	41 (39–44)	39 (37–42)	45 (43–48)	44 (42–47)	45 (42–47)	45 (43–47)	43 (41–45)	43 (41–45)	44 (43–44)
RADT used, % of visits (95% CI)	64 (62–66)	65 (63–67)	65 (63–67)	66 (64–68)	65 (63–67)	69 (66–71)	66 (63–68)	64 (61–66)	62 (60–65)	63 (61–66)	66 (63–68)	67 (65–69)	65 (65–66)
Positive RADTs/all RADTs, % (95% CI)	68 (65–70)	68 (65–70)	68 (65–71)	69 (67–72)	68 (66–71)	66 (63–69)	60 (57–63)	54 (51–57)	56 (53–59)	61 (58–64)	66 (63–68)	68 (65–70)	65 (64–66)
Culture obtained, % of visits (95% CI)	4.8 (4.0–5.9)	5.5 (4.6–6.6)	5.7 (4.8–6.8)	5.7 (4.7–6.9)	6.1 (5.1–7.4)	6.3 (5.3–7.6)	6.2 (5.1–7.5)	8.8 (7.5–10.3)	8.5 (7.2–10.0)	6.4 (5.4–7.6)	6.6 (5.5–7.9)	4.5 (3.7–5.4)	6.2 (5.8–6.5)

*IQR* interquartile range; *CI* confidence interval; *RADT* rapid antigen detection test for group A streptococci

*The numbers are weighted to the length of the month (divided by the number of days and multiplied by 30)

**Table 3:** Summary of visits with a diagnosis of pharyngotonsillitis, per calendar month (2013–2016 combined). Age 0–14 years

	Jan	Feb	Mar	Apr	May	Jun	Jul	Aug	Sep	Oct	Nov	Dec	$\overline{x }$
Mean number of weighted visits*	180	198	187	193	163	169	126	107	111	160	166	202	163
Women, % of visits (95% CI)	56 (52–59)	52 (48–55)	50 (47–54)	51 (47–55)	49 (45–53)	49 (45–53)	51 (46–55)	48 (43–53)	51 (47–56)	54 (50–58)	52 (48–56)	53 (49–56)	51 (50–52)
RADT used, % of visits (95% CI)	69 (65–72)	69 (65–72)	69 (66–72)	71 (68–75)	67 (64–71)	71 (67–74)	67 (63–71)	67 (63–71)	64 (60–68)	66 (62–69)	70 (67–73)	69 (66–72)	68 (67–69)
Positive RADTs/all RADTs, % (95% CI)	80 (77–83)	79 (75–82)	80 (76–83)	85 (81–88)	82 (78–85)	78 (74–82)	73 (68–78)	73 (68–78)	69 (63–73)	72 (67–76)	76 (72–80)	81 (77–84)	78 (77–79)
Culture obtained, % of visits (95% CI)	2.7 (1.7–4.1)	2.8 (1.9–4.3)	3.4 (2.3–4.9)	4.0 (2.7–5.8)	3.6 (2.4–5.2)	3.4 (2.2–5.1)	3.8 (2.5–5.9)	5.2 (3.5–7.7)	4.5 (3.0–6.7)	4.1 (2.8–5.9)	3.1 (2.1–4.7)	1.9 (1.2–3.1)	3.4 (3.0–3.8)

*CI* confidence interval; *RADT* rapid antigen detection test for group A streptococci

*The numbers are weighted to the length of the month (divided by the number of days and multiplied by 30)

**Table 4:** Summary of visits with a diagnosis of pharyngotonsillitis, per calendar month (2013–2016 combined). Age 15–29 years

	Jan	Feb	Mar	Apr	May	Jun	Jul	Aug	Sep	Oct	Nov	Dec	$\overline{x }$
Mean number of weighted visits*	130	120	124	113	112	122	122	143	128	137	126	130	125
Women, % of visits (95% CI)	42 (38–46)	42 (37–46)	34 (30–38)	39 (35–44)	40 (36–45)	35 (31–39)	44 (40–49)	43 (39–47)	43 (39–47)	42 (38–46)	37 (33–42)	39 (35–43)	40 (39–41)
RADT used, % of visits (95% CI)	59 (55–63)	62 (58–66)	61 (57–65)	60 (55–64)	64 (59–68)	67 (63–71)	66 (62–70)	61 (57–65)	61 (57–65)	64 (60–68)	62 (57–66)	62 (58–66)	62 (61–64)
Positive RADTs/all RADTs, % (95% CI)	48 (43–54)	50 (44–55)	49 (44–55)	48 (42–54)	48 (43–54)	50 (44–55)	46 (40–51)	36 (31–41)	40 (35–46)	44 (39–49)	49 (44–55)	45 (40–51)	46 (44–48)
Culture obtained, % of visits (95% CI)	8.0 (6.0–10.6)	8.9 (6.7–11.7)	9.2 (7.0–12.0)	8.6 (6.4–11.5)	9.5 (7.2–12.6)	9.3 (7.1–12.2)	9.7 (7.4–12.6)	10.2 (8.0–12.9)	12.6 (10.1–15.7)	8.0 (6.0–10.5)	11.9 (9.4–14.9)	7.8 (5.8–10.4)	9.5 (8.8–10.2)

*CI* confidence interval; *RADT* rapid antigen detection test for group A streptococci

*The numbers are weighted to the length of the month (divided by the number of days and multiplied by 30)

**Table 5:** Summary of visits with a diagnosis of pharyngotonsillitis, per calendar month (2013–2016 combined). Age 30+ years

	Jan	Feb	Mar	Apr	May	Jun	Jul	Aug	Sep	Oct	Nov	Dec	$\overline{x }$
Mean number of weighted visits*	170	155	155	144	144	156	143	124	104	133	121	178	144
Women, % of visits (95% CI)	40 (36–43)	42 (38–46)	39 (36–43)	37 (33–41)	34 (30–38)	34 (30–37)	41 (37–45)	42 (38–46)	39 (34–43)	37 (33–41)	36 (32–41)	36 (32–39)	38 (37–39)
RADT used, % of visits (95% CI)	63 (60–67)	64 (60–68)	63 (59–67)	65 (61–69)	63 (59–67)	68 (64–72)	63 (59–67)	64 (59–68)	62 (57–66)	60 (56–64)	64 (60–68)	68 (65–72)	64 (63–65)
Positive RADTs/all RADTs, % (95% CI)	67 (63–71)	67 (62–71)	67 (63–72)	67 (62–71)	68 (63–73)	67 (62–71)	61 (56–66)	56 (51–62)	61 (55–66)	66 (60–71)	66 (60–71)	68 (64–72)	65 (64–67)
Culture obtained, % of visits (95% CI)	4.7 (3.4–6.5)	5.9 (4.4–8.1)	5.8 (4.2–7.9)	5.2 (3.7–7.3)	6.4 (4.7–8.7)	6.8 (5.1–9.1)	5.3 (3.7–7.4)	10.4 (8.0–13.3)	7.9 (5.7–10.9)	7.6 (5.7–10.2)	6.0 (4.2–8.5)	4.9 (3.6–6.7)	6.3 (5.7–6.9)

*CI* confidence interval; *RADT* rapid antigen detection test for group A streptococci

*The numbers are weighted to the length of the month (divided by the number of days and multiplied by 30)

### Use of rapid antigen detection tests for group A streptococci

A RADT for GAS was used in 65% (95% CI 65–66) of the visits, totalling 13 817 tests. The proportion of visits where a RADT was used did show a seasonal variation comparing means for all calendar months (p = 0.015). The highest proportion was seen in June (69%, 95% CI 66–71) and the lowest in September (62%, 95% CI 60–65), with a peak-to-low ratio of 1.10 (95% CI 1.05–1.16, p < 0.001). Non-aggregated data are also shown in Additional file 1: Table 2.

### Results of rapid antigen detection tests for group A streptococci

**Fig. 2:**
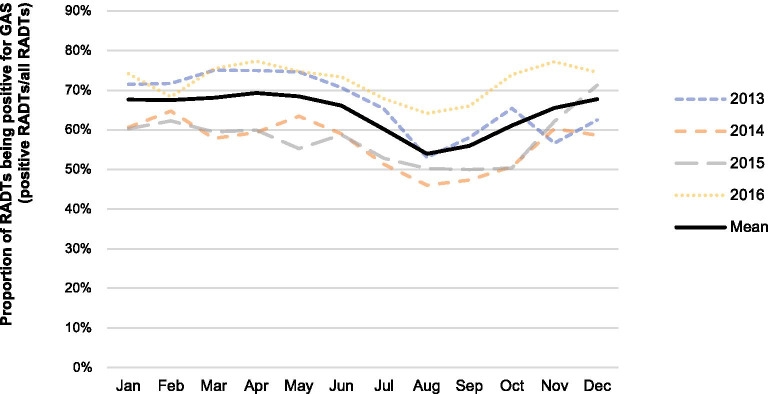
Proportion of RADTs being positive for GAS, at visits resulting in a diagnosis of pharyngotonsillitis. *RADT* rapid antigen detection test; *GAS* group A streptococci

The mean proportion of RADTs being positive for GAS (positive RADTs/all RADTs) was 65% (95% CI 64–66). It showed seasonal variation comparing means for all calendar months (p < 0.001). The highest mean proportion was seen in April (69%, 95% CI 67–72) and the lowest in August (54%, 95% CI 51–57), with a peak-to-low ratio of 1.28 (95% CI 1.20–1.38, p < 0.001). The second lowest mean proportion was seen in September (56%, 95% CI 53–59), with a corresponding ratio (April/September) of 1.24 (95% CI 1.15–1.33, p < 0.001). This seasonal pattern was seen in all age groups (Tables 2, 3, 4, 5). Data from all years are shown in Fig. 2.

### Use of throat cultures

**Fig. 3:**
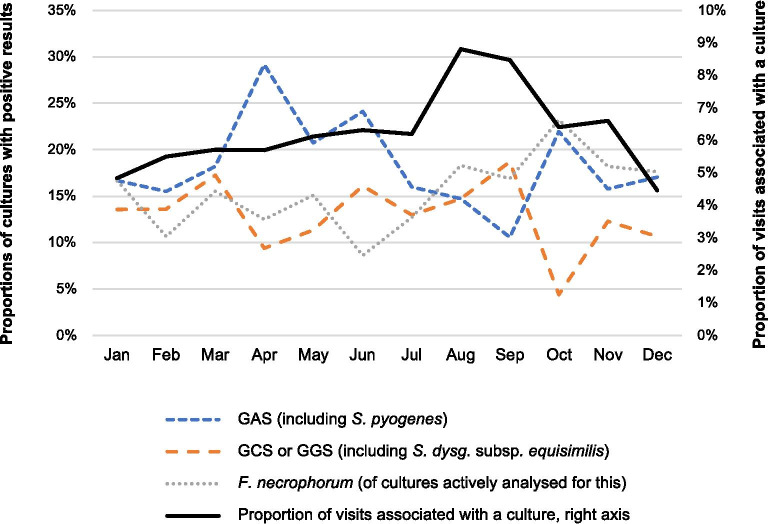
Proportions of cultures with positive results (left axis), obtained from patients with a diagnosis of pharyngotonsillitis (2013–2016 combined). The denominator for group A streptococci (or *S. pyogenes*) and group C or G streptococci (or *S. dysgalactiae* subspecies *equisimilis*) is the total number of cultures obtained, and for *F. necrophorum* the total number of cultures actively targeting this species. The overall variation between calendar months was not statistically significant. The proportion of visits associated with a culture (2013–2016 combined) is shown on a separate axis (right). *RADT* rapid antigen detection test; *GAS* group A streptococci; *GCS* group C streptococci; *GGS* group G streptococci

In total, 1304 throat cultures were obtained from the patient cohort, after exclusion of eight duplicates (more than one culture from the same patient and date). The mean proportion of visits where a culture was obtained was 6.2% (95% CI 5.8–6.5), and when comparing means for all calendar months, it showed significant seasonal variation (p < 0.001). The highest mean proportion was seen in August (8.8%, 95% CI 7.5–10.3) and the lowest in December (4.5%, 95% CI 3.7–5.4), with a peak-to-low ratio of 1.97 (95% CI 1.53–2.55, p < 0.001). The second highest mean proportion was seen in September (8.5%, 95% CI 7.2–10.0), with a corresponding ratio (September/December) of 1.90 (95% CI 1.46–2.46, p < 0.001). The proportions of visits associated with a culture are shown in Fig. 3. There was a negative correlation between the mean proportion of RADTs being positive for GAS (positive RADTs/all RADTs), and the mean proportion of visits associated with a culture in the same calendar month (r = − 0.86).

Among the visits with a RADT being negative for GAS, 14% (95% CI 13–15) resulted in a culture being obtained, compared to 2% (95% CI 1.8–2.3) of the visits with a RADT being positive for GAS. A majority of the cultures was obtained from patients with a RADT being negative for GAS (53%, 95% CI 51–56) or from patients where no RADT had been used on that date (33%, 95% CI 30–36).

Of all the cultures, 52% (95% CI 49–55) were analysed for both beta-haemolytic streptococci and *F. necrophorum* at the laboratory, while the remaining 48% were analysed for beta-haemolytic streptococci only (Additional file 1: Table 3). This proportion did not show any significant seasonal variation.

### Results of throat cultures

Most cultures were negative (61%, 95% CI 58–63), and this proportion did not vary significantly comparing means for all calendar months (p = 0.86). Of all cultures, 18% (95% CI 16–20) were positive for GAS, with the highest mean proportion in April (29%, 95% CI 21–39) and the lowest in September (11%, 95% CI 6–17), but the overall variation between the calendar months, comparing means for all months, was not statistically significant (p = 0.055). GCS or GGS were detected in 13% (95% CI 11–15) of the cultures, with the highest (non-significant) mean proportion in September (19%, 95% CI 13–27). *F. necrophorum* was detected in 16% (95% CI 13–19) of the cultures targeting this species, with the highest (non-significant) mean proportion in October (23%, 95% CI 14–36). Of all positive cultures (n = 513), 21 were positive for more than one of the analysed bacteria. Almost all of these (n = 20) were positive for *F. necrophorum*, and more often in combination with GCS or GGS (n = 14) than with GAS (n = 6). The proportions of positive cultures are shown in Fig. 3.

## Discussion

In this study, more light has been shed on the seasonal variations in the aetiology of pharyngotonsillitis in primary care. As expected, a seasonal variation was seen in the mean rate of visits with a diagnosis of pharyngotonsillitis comparing calendar months, with the highest mean observed in December and the lowest in September. More interestingly, a lower mean rate of visits in August and September coincided with a lower proportion of RADTs being positive for GAS among them, which correlated with a higher proportion of visits associated with a throat culture. The high number of visits and RADTs analysed, during 4 consecutive years, provides strong evidence for a lower likelihood of GAS involvement in August and September than during the rest of the year.

Subgroup analysis indicated a somewhat different visiting behaviour for adolescents and young adults (15–29 years), with a mean peak of visits in August instead of December. However, this age group showed the same pattern of a lower proportion of RADTs being positive for GAS in August and September, thus making age a less likely explanatory factor for this variation in GAS involvement. The peak of visits in August among adolescents and young adults was unexpected, and it remains to be explained what factors are involved in this difference between age groups. Furthermore, men were overrepresented from the age of 15 years and above, which contrasts with what is generally seen in primary care visits [13].

As to other studies of seasonal variations in the aetiology of pharyngotonsillitis, a lower likelihood of GAS involvement among sore throat cases in the northern temperate summer than in the colder winter has previously been hypothesized [9]. The incidence of GAS-associated pharyngotonsillitis in children has also been shown to be higher when temperatures are lower (in a Mediterranean climate), and when the number of school-free days is reduced [14]. Recently, seasonal variations in the percentage of RADT positivity have been reported among children with symptoms of acute pharyngitis in a paediatric hospital setting in Portugal, with a decline in August, similar to our findings [15]. A seasonal pattern has also been suggested for GAS among peritonsillar abscesses, with one study of Danish hospital patients showing a higher prevalence of GAS during the winter and the spring [16].

The findings in our study of a lower likelihood of GAS involvement in pharyngotonsillitis in August and September than during the rest of the year, seem to confirm that a seasonality of aetiology is present. An overall decline in GAS transmission due to summer holidays (mid-June to mid-August in Sweden) and higher temperatures (with less time spent indoors) appear to be plausible factors behind this pattern, but it still needs to be shown why the same factors would not cause a similar decline in other pathogens. Seasonal variations in the prevalence of other beta-haemolytic streptococci in pharyngotonsillitis have previously been found among symptomatic children, with higher non-GAS recovery rates during warm weather [17]. A contrasting pattern has been reported from Hungary, with a peak incidence of GCS/GGS in pharyngeal isolates during the colder months (January–March) [18]. As to virus-associated cases of sore throat in children, these have been shown to be more common during the Scottish winter [19].

For comparison, seasonal variations in Swedish cases of invasive GAS infection have been described in a report from the Public Health Agency of Sweden, with the incidence being at its highest in the 1st months of the year [20]. Similar reports of invasive infections, with peak incidence of invasive GAS in the winter (December–February) have been reported both from Norway and Hungary, with contrasting incidence peaks of invasive GCS/GGS in the summer (June–August) [18, 21].

Given the limited number of cultures in our study, no conclusions could be made from their results with regard to seasonal variations. The low number of cultures was expected, since the current guidelines do not encourage throat cultures as part of routine diagnosis. Hence, they can be thought of as actively selected cases (for instance treatment failure, relapsing episodes, unexpected clinical presentations or RADT results). It is still unclear whether the decline in GAS in August and September is offset by non-GAS bacteria or if other pathogens are involved. In our study, we did not detect any significant variation between calendar months regarding the proportion of negative cultures.

Another factor, potentially obscuring patterns of seasonal variations in the aetiology, is the seasonal variations in the asymptomatic pharyngeal colonization by potential pathogens. Asymptomatic colonization by GAS has been shown to vary with age, time and concurrent infections in the environment [3]. A small, previous study has also indicated higher prevalence of asymptomatic GAS during the Swedish summer (mid-July to mid-September) than during the winter (mid-January to mid-February) [9]. In the same study, no significant difference was seen in the prevalence of GAS between symptomatic and asymptomatic children < 16 years of age during the summer, which could question the overall applicability of RADTs during this time of the year. However, other studies have indicated the opposite, that the asymptomatic prevalence of GAS and other beta-haemolytic streptococci is independent of season [22, 23].

Obviously, GAS are not the only pathogens causing pharyngotonsillitis, but the significance of non-GAS bacteria and other pathogens needs to be better understood. GCS and GGS are more commonly found among pharyngotonsillitis patients than in asymptomatic controls [8], and GCS have been thought to be of more relevance than GGS [3], but the pathogenic importance of GCS and GGS in pharyngotonsillitis is much less clear than that of GAS [7, 8, 24]. *F. necrophorum* too is more commonly found among patients with pharyngotonsillitis than in asymptomatic controls [8], and more so among teenagers and young adults [11]. However, there is yet no strong evidence for causality, and neither is it known if antibiotic treatment can alleviate symptoms or reduce the risk of complications [25], such as development of the rare Lemierre’s syndrome [26]. Thus, it remains unclear to what extent symptomatic non-GAS cases could benefit from antibiotic treatment. In general, the absolute benefits of antibiotics for sore throats are considered modest, and the number needed to treat is high, to prevent suppurative and non-suppurative complications in high-income countries [27].

The study has methodological limitations. For example, no validation of diagnoses was made, as no individual EMRs were read. Several previous studies have suggested that the adherence to diagnostic guidelines in clinical practice is low [28, 29]. It could be suspected that the outcome of RADTs for GAS among patients with a sore throat is in practice affecting the choice of diagnosis, altering the “true” proportion of positive/negative RADTs among patients with pharyngotonsillitis. However, our study does not describe the general population of sore throats, but one of visits resulting in a diagnosis of acute tonsillitis or pharyngitis. Because of the Swedish health care system, with publicly financed primary care to everyone, and with compulsory diagnosing of all visits, our data can be expected to represent the vast majority of diagnoses of pharyngotonsillitis in our county during the period studied. Another limitation is our time span for duplicate exclusion. Choosing a longer time span, aiming at finding disease episodes rather than visits, would have been an alternative approach. This could have reduced the risk of overestimation of cases due to “second opinions”, for instance after getting a negative RADT. However, setting an optimal time span for what to exclude would not be easy, and a longer time span could have led to exclusion of cultures representing treatment failure that we wanted to find. Thus, we chose to accept this risk of “dilution”, which should be kept in mind also when interpreting the proportion of visits with RADT being used and the proportion of RADTs being positive.

## Conclusions

Among the visits with a diagnosis of pharyngotonsillitis, variation between calendar months was seen as to likelihood of GAS involvement. A lower proportion of RADTs being positive for GAS was seen in August and September, correlating with a higher use of throat cultures. The role of group A streptococci in pharyngotonsillitis in Sweden appears to be less prominent in August and September than during the rest of the year.

## Supplementary Information

**Additional file 1: Table 1.** Number of visits in primary care resulting in a diagnosis of pharyngotonsillitis (all ages). **Table 2.** Number of rapid antigen detection tests for group A streptococci used at visits with a diagnosis of pharyngotonsillitis. **Table 3.** Number of throat cultures (and positive results) at visits with a diagnosis of pharyngotonsillitis.

## Data Availability

The datasets used and/or analysed during the current study are available from the corresponding author on reasonable request.

## Abbreviations

CI: Confidence interval
EMR: Electronic medical record
GAS/GCS/GGS: Group A/C/G streptococci
ICD-10: International classification of diseases, tenth revision
IQR: Interquartile range
PHCC: Primary health care centre
RADT: Rapid antigen detection test
