# Supplementary material for: Seasonal variations in use and outcome of rapid antigen detection tests and cultures in pharyngotonsillitis: a register study in primary care
Source: BMC Infect Dis. 2021 Oct 26;21:1104. doi: 10.1186/s12879-021-06774-5 (PMC8549259; doi:10.1186/s12879-021-06774-5)
Supplement: Supplementary file 1 — Additional file 1: Table 1. Number of visits in primary care resulting in a diagnosis of pharyngotonsillitis (all ages). Table 2. Number of rapid antigen detection tests for group A streptococci used at visits with a diagnosis of pharyngotonsillitis. Table 3. Number of throat cultures (and positive results) at visits with a diagnosis of pharyngotonsillitis. [file 12879_2021_6774_MOESM1_ESM.docx]

# Seasonal variations in use and outcome of rapid antigen detection tests and cultures in pharyngotonsillitis ­– a register study in primary care

### Additional tables

**Additional Table 1**. Number of visits in primary care resulting in a diagnosis of pharyngotonsillitis (all ages).

| **Year** | | | **Month** | | | | | | | | | | | | | | | | | | | | | | |  | **Total** | | |  |
| --- | --- | --- | --- | --- | --- | --- | --- | --- | --- | --- | --- | --- | --- | --- | --- | --- | --- | --- | --- | --- | --- | --- | --- | --- | --- | --- | --- | --- | --- | --- |
|  |  |  | Jan | (%) | Feb | (%) | Mar | (%) | Apr | (%) | May | (%) | Jun | (%) | Jul | (%) | Aug | (%) | Sep | (%) | Oct | (%) | Nov | (%) | Dec | (%) | |  | (%) | |
| **2013** |  | Women | 303 | (44) | 266 | (44) | 258 | (42) | 250 | (44) | 220 | (42) | 176 | (38) | 238 | (50) | 192 | (45) | 157 | (46) | 223 | (48) | 171 | (43) | 209 | (43) | | 2 663 | (44) | |
|  |  | Men | 390 | (56) | 343 | (56) | 354 | (58) | 317 | (56) | 309 | (58) | 286 | (62) | 238 | (50) | 231 | (55) | 183 | (54) | 242 | (52) | 225 | (57) | 279 | (57) | | 3 397 | (56) | |
|  |  | Total | 693 |  | 609 |  | 612 |  | 567 |  | 529 |  | 462 |  | 476 |  | 423 |  | 340 |  | 465 |  | 396 |  | 488 |  | | 6 060 |  | |
| **2014** |  | Women | 210 | (49) | 187 | (41) | 184 | (42) | 148 | (42) | 194 | (47) | 181 | (42) | 170 | (43) | 167 | (45) | 153 | (42) | 196 | (43) | 173 | (45) | 218 | (48) | | 2 181 | (44) | |
|  |  | Men | 216 | (51) | 265 | (59) | 250 | (58) | 201 | (58) | 222 | (53) | 251 | (58) | 228 | (57) | 201 | (55) | 213 | (58) | 256 | (57) | 210 | (55) | 240 | (52) | | 2 753 | (56) | |
|  | Total | | 426 |  | 452 |  | 434 |  | 349 |  | 416 |  | 432 |  | 398 |  | 368 |  | 366 |  | 452 |  | 383 |  | 458 |  | | 4 934 |  | |
| **2015** |  | Women | 181 | (46) | 191 | (50) | 173 | (42) | 159 | (42) | 137 | (37) | 163 | (39) | 142 | (41) | 147 | (42) | 150 | (44) | 173 | (44) | 167 | (41) | 184 | (40) | | 1 967 | (42) | |
|  |  | Men | 213 | (54) | 188 | (50) | 242 | (58) | 217 | (58) | 229 | (63) | 254 | (61) | 202 | (59) | 201 | (58) | 190 | (56) | 223 | (56) | 240 | (59) | 273 | (60) | | 2 672 | (58) | |
|  | Total | | 394 |  | 379 |  | 415 |  | 376 |  | 366 |  | 417 |  | 344 |  | 348 |  | 340 |  | 396 |  | 407 |  | 457 |  | | 4 639 |  | |
| **2016** |  | Women | 221 | (47) | 212 | (49) | 197 | (43) | 164 | (42) | 166 | (40) | 176 | (38) | 179 | (45) | 174 | (43) | 187 | (46) | 206 | (44) | 232 | (43) | 301 | (43) | | 2 415 | (44) | |
|  |  | Men | 247 | (53) | 222 | (51) | 265 | (57) | 230 | (58) | 253 | (60) | 285 | (62) | 216 | (55) | 231 | (57) | 218 | (54) | 260 | (56) | 307 | (57) | 402 | (57) | | 3 136 | (56) | |
|  | Total | | 468 |  | 434 |  | 462 |  | 394 |  | 419 |  | 461 |  | 395 |  | 405 |  | 405 |  | 466 |  | 539 |  | 703 |  | | 5 551 |  | |
| **Total** |  | Women | 915 | (46) | 856 | (46) | 812 | (42) | 721 | (43) | 717 | (41) | 696 | (39) | 729 | (45) | 680 | (44) | 647 | (45) | 798 | (45) | 743 | (43) | 912 | (43) | | 9 226 | (44) | |
|  |  | Men | 1 066 | (54) | 1 018 | (54) | 1 111 | (58) | 965 | (57) | 1 013 | (59) | 1 076 | (61) | 884 | (55) | 864 | (56) | 804 | (55) | 981 | (55) | 982 | (57) | 1 194 | (57) | | 11 958 | (56) | |
|  | Total | | 1 981 |  | 1 874 |  | 1 923 |  | 1 686 |  | 1 730 |  | 1 772 |  | 1 613 |  | 1 544 |  | 1 451 |  | 1 779 |  | 1 725 |  | 2 106 |  | | 21 184 |  | |

**Additional Table 2.** Number of rapid antigen detection tests for group A streptococci used at visits with a diagnosis of pharyngotonsillitis.

| **Year** | | | | **Month** | | | | | | | | | | | |  | | | | | | | | | | | | **Total** | |  |
| --- | --- | --- | --- | --- | --- | --- | --- | --- | --- | --- | --- | --- | --- | --- | --- | --- | --- | --- | --- | --- | --- | --- | --- | --- | --- | --- | --- | --- | --- | --- |
|  |  |  |  | Jan | (%) | Feb | (%) | Mar | (%) | Apr | (%) | May | (%) | Jun | (%) | Jul | (%) | Aug | (%) | Sep | (%) | Oct | (%) | Nov | (%) | Dec | (%) |  | (%) | |
| **2013** | Neg |  | | 133 | (28) | 117 | (28) | 100 | (25) | 96 | (25) | 92 | (25) | 95 | (29) | 108 | (35) | 122 | (47) | 86 | (42) | 107 | (35) | 114 | (43) | 117 | (38) | 1 287 | (32) | |
|  | Pos | | | 334 | (72) | 297 | (72) | 301 | (75) | 288 | (75) | 271 | (75) | 229 | (71) | 204 | (65) | 138 | (53) | 119 | (58) | 203 | (65) | 149 | (57) | 195 | (63) | 2 728 | (68) | |
|  | Total | | | 467 |  | 414 |  | 401 |  | 384 |  | 363 |  | 324 |  | 312 |  | 260 |  | 205 |  | 310 |  | 263 |  | 312 |  | 4 015 |  | |
| **2014** | Neg |  | | 105 | (39) | 99 | (35) | 112 | (42) | 86 | (41) | 92 | (37) | 115 | (41) | 115 | (49) | 130 | (54) | 111 | (53) | 132 | (49) | 93 | (40) | 126 | (41) | 1 316 | (43) | |
|  | Pos |  | | 162 | (61) | 182 | (65) | 154 | (58) | 126 | (59) | 160 | (63) | 166 | (59) | 121 | (51) | 111 | (46) | 100 | (47) | 136 | (51) | 141 | (60) | 179 | (59) | 1 738 | (57) | |
|  | Total | | | 267 |  | 281 |  | 266 |  | 212 |  | 252 |  | 281 |  | 236 |  | 241 |  | 211 |  | 268 |  | 234 |  | 305 |  | 3 054 |  | |
| **2015** | Neg |  | | 98 | (40) | 89 | (38) | 111 | (41) | 97 | (40) | 96 | (45) | 108 | (41) | 108 | (47) | 99 | (50) | 108 | (50) | 118 | (50) | 96 | (38) | 87 | (29) | 1 215 | (42) | |
|  | Pos |  | | 149 | (60) | 147 | (62) | 163 | (59) | 145 | (60) | 119 | (55) | 154 | (59) | 121 | (53) | 100 | (50) | 108 | (50) | 120 | (50) | 158 | (62) | 216 | (71) | 1 700 | (58) | |
|  | Total | | | 247 |  | 236 |  | 274 |  | 242 |  | 215 |  | 262 |  | 229 |  | 199 |  | 216 |  | 238 |  | 254 |  | 303 |  | 2 915 |  | |
| **2016** | Neg |  | | 76 | (26) | 93 | (32) | 76 | (25) | 62 | (23) | 74 | (25) | 93 | (27) | 90 | (32) | 101 | (36) | 92 | (34) | 81 | (26) | 87 | (23) | 125 | (25) | 1 050 | (27) | |
|  | Pos |  | | 219 | (74) | 201 | (68) | 234 | (75) | 212 | (77) | 219 | (75) | 256 | (73) | 190 | (68) | 181 | (64) | 179 | (66) | 230 | (74) | 296 | (77) | 366 | (75) | 2 783 | (73) | |
|  | Total | | | 295 |  | 294 |  | 310 |  | 274 |  | 293 |  | 349 |  | 280 |  | 282 |  | 271 |  | 311 |  | 383 |  | 491 |  | 3 833 |  | |
| **Total** | Neg |  | | 412 | (32) | 398 | (32) | 399 | (32) | 341 | (31) | 354 | (32) | 411 | (34) | 421 | (40) | 452 | (46) | 397 | (44) | 438 | (39) | 390 | (34) | 455 | (32) | 4 868 | (35) | |
|  | Pos |  | | 864 | (68) | 827 | (68) | 852 | (68) | 771 | (69) | 769 | (68) | 805 | (66) | 636 | (60) | 530 | (54) | 506 | (56) | 689 | (61) | 744 | (66) | 956 | (68) | 8 949 | (65) | |
|  | Total | | 1 276 | | 1 225 | | 1 251 | | 1 112 | | 1 123 | | 1 216 | | 1 057 | | 982 | | 903 | | 1 127 | | 1 134 | | 1 411 | | 13 817 | |  | |

**Additional Table 3**. Number of throat cultures (and positive results) at visits with a diagnosis of pharyngotonsillitis.

| Year | Month | Total |
| --- | --- | --- |

|  |  | Jan | Feb | Mar | Apr | May | Jun | Jul | Aug | Sep | Oct | Nov | Dec |  |
| --- | --- | --- | --- | --- | --- | --- | --- | --- | --- | --- | --- | --- | --- | --- |
| **2013** | All cultures | 24 | 35 | 31 | 27 | 29 | 24 | 24 | 35 | 29 | 30 | 26 | 14 | 328 |
|  | Cultures actively analysed for *F. necrophorum* | 9 | 13 | 11 | 14 | 15 | 9 | 11 | 10 | 13 | 14 | 11 | 5 | 135 |
|  | GAS *(S. pyogenes)* | 5 | 4 | 6 | 8 | 9 | 5 | 6 | 5 | 3 | 8 | 5 |  | 64 |
|  | GCS | 1 | 3 | 1 | 1 | 1 | 3 | 3 | 4 | 4 | 1 |  |  | 22 |
|  | GGS | 3 | 2 | 5 | 1 | 1 |  | 2 | 1 | 1 |  | 1 |  | 17 |
|  | *S. dysgalactiae* subsp*. equisimilis* |  |  |  |  |  |  |  |  |  |  | 1 | 3 | 4 |
|  | Subtotal of GCS + GGS + *S. dysgalactiae* subsp*. equisimilis* | 4 | 5 | 6 | 2 | 2 | 3 | 5 | 5 | 5 | 1 | 2 | 3 | 43 |
|  | *F. necrophorum* | 1 | 1 | 1 | 1 | 3 |  | 2 | 1 | 2 | 4 | 2 | 1 | 19 |
|  | Subtotal of positive cultures | 10 | 10 | 13 | 11 | 14 | 8 | 13 | 11 | 10 | 13 | 9 | 4 | 126 |
| **2014** | All cultures | 29 | 35 | 31 | 16 | 29 | 35 | 34 | 39 | 31 | 40 | 28 | 27 | 374 |
|  | Cultures actively analysed for *F. necrophorum* | 16 | 18 | 17 | 11 | 15 | 16 | 21 | 20 | 19 | 21 | 15 | 15 | 204 |
|  | *S. pyogenes* | 6 | 1 | 5 | 3 | 5 | 7 | 3 | 7 | 3 | 9 | 5 | 5 | 59 |
|  | *S. dysgalactiae* subsp*. equisimilis* | 4 | 5 | 3 | 2 | 6 | 3 | 2 | 5 | 7 | 2 | 4 | 3 | 46 |
|  | *F. necrophorum* | 3 | 2 | 4 | 3 | 3 | 2 | 2 | 3 | 2 | 3 | 4 | 4 | 35 |
|  | Subtotal of positive cultures | 13 | 8 | 12 | 8 | 14 | 12 | 7 | 15 | 12 | 14 | 13 | 12 | 140 |
| **2015** | All cultures | 30 | 18 | 19 | 25 | 26 | 26 | 18 | 34 | 30 | 26 | 30 | 28 | 310 |
|  | Cultures actively analysed for *F. necrophorum* | 14 | 6 | 11 | 11 | 15 | 15 | 10 | 17 | 18 | 9 | 18 | 19 | 163 |
|  | *S. pyogenes* | 2 | 6 | 3 | 7 | 4 | 7 | 2 | 5 | 5 | 5 | 6 | 5 | 57 |
|  | *S. dysgalactiae* subsp*. equisimilis* | 4 | 3 | 3 | 1 | 1 | 5 | 4 | 5 | 5 | 1 | 4 | 3 | 39 |
|  | *F. necrophorum* | 3 |  | 1 | 1 | 1 | 2 | 2 | 3 | 4 | 3 | 4 | 3 | 27 |
|  | Subtotal of positive cultures | 9 | 9 | 7 | 9 | 6 | 14 | 8 | 13 | 14 | 9 | 14 | 11 | 123 |
| **2016** | All cultures | 13 | 15 | 29 | 28 | 22 | 27 | 24 | 28 | 33 | 18 | 30 | 25 | 292 |
|  | Cultures actively analysed for *F. necrophorum* | 9 | 10 | 19 | 20 | 8 | 18 | 13 | 13 | 21 | 12 | 22 | 12 | 177 |
|  | *S. pyogenes* | 3 | 5 | 6 | 10 | 4 | 8 | 5 | 3 | 2 | 3 | 2 | 6 | 57 |
|  | *S. dysgalactiae* subsp*. equisimilis* | 1 | 1 | 7 | 4 | 3 | 7 | 2 | 5 | 6 | 1 | 4 | 1 | 42 |
|  | *F. necrophorum* | 1 | 2 | 3 | 2 | 1 | 1 | 1 | 4 | 4 | 3 | 2 | 1 | 25 |
|  | Subtotal of positive cultures | 5 | 8 | 16 | 16 | 8 | 16 | 8 | 12 | 12 | 7 | 8 | 8 | 124 |
| **Total** | All cultures | 96 | 103 | 110 | 96 | 106 | 112 | 100 | 136 | 123 | 114 | 114 | 94 | 1 304 |
|  | Cultures actively analysed for *F. necrophorum* | 48 | 47 | 58 | 56 | 53 | 58 | 55 | 60 | 71 | 56 | 66 | 51 | 679 |
|  | *S. pyogenes* (GAS) | 16 | 16 | 20 | 28 | 22 | 27 | 16 | 20 | 13 | 25 | 18 | 16 | 237 |
|  | *S. dysgalactiae* subsp*. equisimilis* (GCS + GGS) | 13 | 14 | 19 | 9 | 12 | 18 | 13 | 20 | 23 | 5 | 14 | 10 | 170 |
|  | *F. necrophorum* | 8 | 5 | 9 | 7 | 8 | 5 | 7 | 11 | 12 | 13 | 12 | 9 | 106 |
|  | Total of positive cultures | 37 | 35 | 48 | 44 | 42 | 50 | 36 | 51 | 48 | 43 | 44 | 35 | 513 |

*F. necrophorum*: *Fusobacterium necrophorum*; *S. pyogenes*: *Streptococcus pyogenes*; GAS: Group A streptococci; GCS: Group C streptococci; GGS: Group G streptococci; *S. dysgalactiae* subsp*. equisimilis*: *Streptococcus dysgalactiae* subspecies *equisimilis*. In late 2013, the microbiological laboratory switched from Lancefield classification to binomial nomenclature. For the analyses in this article, we chose to treat group A beta-hemolytic streptococci (GAS) and *S. pyogenes* as interchangeable, and similarly, to collectively treat group C (GCS) and G (GGS) streptococci as equivalent to *S. dysgalactiae* subsp*. equisimilis*.
